# Inhibitory Effects of *Caulerpa racemosa*, *Ulva intestinalis*, and *Lobophora challengeriae* on Tyrosinase Activity and α-MSH-Induced Melanogenesis in B16F10 Melanoma Cells

**DOI:** 10.3390/life13040934

**Published:** 2023-04-03

**Authors:** Pradtana Choosuwan, Jantana Praiboon, Korawinwich Boonpisuttinant, Anirut Klomjit, Narongrit Muangmai, Rapeeporn Ruangchuay, Anong Chirapart

**Affiliations:** 1 Algal Bioresources Research Center, Department of Fishery Biology, Faculty of Fisheries, Kasetsart University, Chatuchak, Bangkok 10900, Thailand; 2 Innovative Natural Products from Thai Wisdoms (INPTW), Faculty of Integrative Medicine, Rajamangala University of Technology Thanyaburi, Pathumthani 12130, Thailand; 3 Division of Fishery Technology, Faculty of Science and Technology, Prince of Songkla University, Pattani 94000, Thailand

**Keywords:** hyperpigmentation, intracellular tyrosinase, melanin, seaweed, whitening agent

## Abstract

Melanogenesis involves a synthesis of melanin pigment and is regulated by tyrosinase. The addition of whitening agents with tyrosinase-inhibiting properties in cosmetics is becoming increasingly important. In this study, the ethanolic extracts from twelve seaweeds were assessed for tyrosinase-inhibiting activity using mushroom tyrosinase and melanin synthesis in B16F10 melanoma cells. The highest mushroom tyrosinase inhibition (IC_50_) was observed with *Lobophora challengeriae* (0.15 ± 0.01 mg mL^−1^); treatment was more effective than kojic acid (IC_50_ = 0.35 ± 0.05 mg mL^−1^), a well-known tyrosinase inhibitor. Three seaweeds, *Caulerpa racemosa*, *Ulva intestinalis*, and *L. challengeriae*, were further investigated for their ability to reduce melanogenesis in B16F10 cells. The ethanolic extracts of *C. racemosa*, *U. intestinalis*, and *L. challengeriae* showed inhibitory effects by reducing melanin and intracellular tyrosinase levels in B16F10 cells treated with α-melanocyte stimulating hormone in a dose-dependent manner. *C. racemosa* (33.71%) and *L. challengeriae* (36.14%) at 25 µg mL^−1^ reduced melanin production comparable to that of kojic acid (36.18%). *L. challengeriae* showed a stronger inhibition of intracellular tyrosinase (decreased from 165.23% to 46.30%) than kojic acid (to 72.50%). Thus, ethanolic extracts from *C. racemosa*, *U. intestinalis*, and *L. challengeriae* can be good sources of natural tyrosinase inhibitors and therapeutic or cosmetic agents in the future.

## 1. Introduction

The melanin content of mammals is the main determining factor of skin and hair colors [1]. In addition, melanin protects skin cells from ultraviolet (UV) radiation damage by absorbing UV radiation and removing reactive oxygen species (ROS) [2,3]. The overproduction of melanin is one of the main causes of melasma, freckles, dark spots, and post-inflammatory melanoderma [4]. Melanin is produced from epidermal melanocytes along with basal keratinocytes, and melanocytes synthesize melanin through a process called melanogenesis. Tyrosinase-related protein-1 (TRP-1), TRP-2, and tyrosinase are enzymes that regulate the process of melanin production [5]. Tyrosinase (enzyme commission number (EC) 1.14.18.1) is the rate-limiting enzyme that plays an important role in melanogenesis [6]. This enzyme is mainly involved in the initial steps of melanogenesis; it catalyzes the hydroxylation of L-tyrosine to L-DOPA and converts L-DOPA to dopaquinone by oxidation. Dopaquinone can be transformed to dopachrome in the production of eumelanin, and dopaquinone itself can also be converted to cysteinyl dopa to produce pheomelanin [7,8]. Tyrosinase inhibition is one of the most common methods for achieving skin hypopigmentation [9]. In addition, tyrosinase inhibitors may be of clinical benefit in the treatment of skin cancer and some dermatologic conditions associated with melanin hyperpigmentation [10].

Tyrosinase inhibitors are known from both natural and synthetic sources. Although many tyrosinase inhibitors are available today, only a few can be used in cosmetics because of their side effects. Hydroquinone, kojic acid, and arbutin are known inhibitors used for the treatment of hyperpigmentation disorders. Some studies have emphasized that hydroquinone can cause DNA damage, mutations and have reported various side effects, including contact dermatitis, leukoderma, transient erythema, burning, prickling sensations, and ochronosis [1,11,12,13]. Kojic acid has also been banned in some countries due to side effects and concerns about mutagenicity [14]. Although arbutin is a natural product, it affects the gene expression level of the skin [15]. Safe and effective tyrosinase inhibitors from natural resources thus continue to attract attention, and in recent years seaweed has become an increasingly promising potential source of tyrosinase inhibitors.

Several bioactive compounds from seaweed are considered excellent natural extracts that can be used as ingredients in the development of cosmetic and pharmaceutical products [3,16,17]. According to previous studies, seaweeds are promising sources of novel tyrosinase inhibitors because of their anti-melanogenic effects, especially brown seaweeds belonging to the genera *Ecklonia*, *Laminaria*, *Sargassum*, and *Undaria* [9,10,18,19,20,21,22]. Some studies have been reported on red seaweed [18,23,24] and green seaweed [19,25,26].

*Caulerpa racemosa* (Forsskål) J. Agardh (1873) [27], *Ulva intestinalis* Linnaeus (1753) [28], and *Lobophora challengeriae* C.W. Vieira (2019) [29] are seaweeds native to the southern Pacific Ocean and are typically distributed along the coasts of Thailand [30,31]. Currently, *C. racemosa* and *U. intestinalis* are green algae that can be produced in large quantities on farms and are used mainly as food or feed [32,33]. The investigation of high-value substances from these seaweeds for nutraceuticals, cosmeceuticals, and pharmaceuticals is still ongoing. *Lobophora* is a brown seaweed that has been reported to have a wide range of bioactivities, including antioxidative, antibacterial, antiviral, and antitumoral activities [34]. As far as is known, this is the first investigation on the inhibitory effect of extracts from *U. intestinalis* and *L. challengeriae* on melanin synthesis in B16F10 cells. Accordingly, the present study aimed to explore the inhibitory effect of seaweed extract using a mushroom tyrosinase system, which is commonly used to test and screen potential tyrosinase inhibitors. The ability of seaweed extract to inhibit melanin production and intracellular tyrosinase activity in B16F10 cells was also tested.

## 2. Materials and Methods

### 2.1. Seaweed

Twelve samples of seaweed were collected in the coastal regions of Thailand from May 2016 to August 2017 (Table 1). After collection, the sands, salts, and epiphytes were removed with fresh water. Seaweed was kept at −20 °C for further analysis. All seaweed samples were identified at the Algal Bioresources Research Center of the Department of Fishery Biology at Kasetsart University.

### 2.2. Seaweed Extract Preparation

The samples were extracted with 95% ethanol (1:10, *w/v*) for 24 h at room temperature. The extract was filtered with Whatman No. 2 filter paper. The residues were extracted two additional times under the same conditions. The resulting filtrate was evaporated at 40 °C under reduced pressure in a rotary evaporator (BUCHI rotavapor R-200) until crude extract was obtained. The extract was redissolved with 95% ethanol, filtered with a 0.45 μm polytetrafluoroethylene (PTFE) syringe filter, blown with N_2_, and stored at −20 °C until further analysis.

### 2.3. Determination of Total Phenolic Contents (TPC)

The TPC of seaweed extracts was analyzed according to the method of Park et al. [42] with slight modifications. Briefly, 100 µL of the sample solution (10 mg mL^−1^) was added to 750 μL of 10% Folin–Ciocalteu solution. After 5 min, 750 μL of 6% Na_2_CO_3_ was added, and the mixture was incubated at room temperature for 30 min in the dark. The absorbance of the samples was measured at 750 nm by a microplate reader (EZ Read 200 microplate reader). The TPC of each extract was then calculated based on a gallic acid standard curve and is reported as milligrams of the gallic acid equivalent (GAE) per gram of the extract.

### 2.4. Mushroom Tyrosinase Assay

The inhibitory effects of seaweed extracts were investigated according to the method described by Chan et al. [43] with slight modifications. The tyrosinase activity was determined using 3,4-dihydroxy-l-phenylalanine (L-DOPA) as a substrate. Briefly, 40 µL of 500 units mL^−1^ mushroom tyrosinase (Sigma, St. Louis, MO, USA) in 0.1 M phosphate buffer (pH 6.8), and 40 µL of various concentrations of the extract were added to each well of a 96-well plate. After 10 min of incubation at room temperature, 80 µL of 2.5 mM L-DOPA in 0.1 M phosphate buffer (pH 6.8) was added to each well and incubated for 30 min at 37 °C. Kojic acid was used as a positive control. The absorbance of samples at 475 nm was measured using a 96-well microplate reader, and results are expressed as a percentage of the control value (without seaweed extract).

### 2.5. Melanocyte Cell Culture

The B16F10 murine melanoma cell line was obtained from the American Type Culture Collection (ATCC, Manassas, VA, USA) and cultured in Dulbecco’s modified Eagle’s medium (DMEM) with 10% fetal bovine serum, 100 units mL^−1^ penicillin, and 100 units mL^−1^ streptomycin at 37 °C in a humidified atmosphere containing 5% CO_2_. Cells were subcultured every 3–4 days. The cells were harvested by trypsinization and counted by a hemocytometer.

### 2.6. Cell Viability Assay

The cytotoxicity of seaweed extracts on B16F10 melanoma cells was determined using a 3-(4,5-dimethylthiazol-2yl)-2,5-diphenyltetrazolium bromide (MTT) assay according to the method of Boonpisuttinant et al. [44] with slight modification. The cells (3 × 10^4^ cells well^−1^) were seeded in 96-well plates with culture media and allowed to adhere overnight at 37 °C in a 5% CO_2_ incubator. Cells were treated with various concentrations of extracts or kojic acid and incubated for an additional 72 h. After treatment, the cells were washed three times with phosphate buffer (pH 7.0), and 100 μL of MTT solution (5 mg mL^−1^ in PBS) was added to each well and incubated at 37 °C for 1 h. After incubation, the MTT solution was removed, and the formazan product in each well was dissolved by adding 100 μL of dimethyl sulfoxide (DMSO). The mixture was incubated at 37 °C for 10 min, and the absorbance was measured at 560 nm using a microplate reader (Expert Plus Microplate Reader, Biochrome, Ltd., Cambridge, UK).

### 2.7. Measurement of Cellular Melanin Contents

The melanin content in B16F10 melanoma cells was measured according to the method of Chan et al. [43] with slight modifications. In brief, cells (2 × 10^5^ cells well^−1^) were suspended in 1 mL of media in 12-well plates and allowed to adhere for 24 h at 37 °C under a 5% CO_2_ atmosphere. Cells were exposed to various concentrations of seaweed extracts and kojic acid (2.5–25 μg mL^−1^) for 72 h in the presence or absence of 100 nM α-melanocyte stimulating hormone (α-MSH), and were washed with PBS (pH 7.0). Cell pellets were lysed in 100 mL of 1 M NaOH at 60 °C for 1 h. The supernatant was measured at 450 nm with a microplate reader, and melanin content was expressed as a percentage change from the α-MSH-treated cells. Cell changes were also observed under a microscope.

### 2.8. Intracellular Tyrosinase Activity Assay

The intracellular tyrosinase activity in B16F10 melanoma cells was determined according to the method of Wang et al. [45] with slight modifications. The cells (2 × 10^5^ cells well^−1^) were seeded in 6-well plates and cultured for 24 h. Cells were exposed to various concentrations of seaweed extracts and kojic acid (2.5, 5, 10, and 25 μg mL^−1^) for 72 h in the presence or absence of 100 nM α-MSH and then washed with PBS (pH 7.0). Cells’ pellets were collected after treatment with trypsin-EDTA and centrifuged at 12,000 rpm for 5 min. The pellets were lysed with 0.1 mM protease inhibitor cocktail and 1% Triton X-100. The lysates were frozen/thawed three times and then centrifuged at 10,000 rpm for 10 min. The supernatant was used for the measurement of tyrosinase activity. The reaction mixture, consisting of 80 µL of cell supernatant and 80 µL of 10 mM L-DOPA, was placed in a 96-well plate and incubated at 37 °C for 1 h. The absorbance was measured at 475 nm with a microplate reader, and the results are expressed as a percentage change from the value of α-MSH-treated cells.

### 2.9. Statistical Analysis

The data are presented as the mean ± standard deviation (SD). All experiments were conducted in triplicate (n = 3). The assessment of statistical difference between mean values was performed with IBM SPSS Statistics 28 software using one-way ANOVA followed by Duncan’s multiple range test. *p* values less than 0.05 (*p* < 0.05) were considered statistically significant. Pearson correlation analysis was applied to determine the relationship between phenolic content and anti-tyrosinase or anti-melanogenic activity. Half-maximal inhibitory concentrations (IC_50_) were calculated using Sigma Plot 12.0 software.

## 3. Results

### 3.1. The Extraction Yields and Total Phenolic Content of Seaweed Extracts

**Figure 1:**
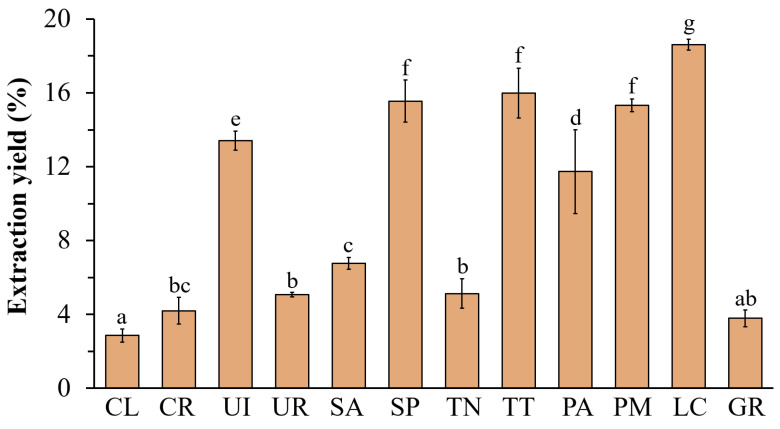
The extraction yields of ethanolic extracts were calculated based on the sample dry weight. The values are expressed as mean± SD (n = 3). Significant differences are indicated by different letters (*p* < 0.05).

The extraction yields of the ethanolic extract from twelve seaweed samples are shown in Figure 1. The highest yield was found in the brown seaweed *L. challengeriae* (LC) (18.61 ± 0.29%), followed by *Sargassum polycystum* (SP), *Turbinaria conoides* (TT), and *Padina minor* (PM), with extraction yields of 15.55 ± 1.14%, 15.99 ± 1.35%, and 15.33 ± 0.35%, respectively. Moreover, *U. intestinalis* (UI) showed the highest yield among the green seaweeds (13.41 ± 0.51%). The extract yield of the red seaweed *Gracilaria dura* (GR) was 3.79 ± 0.46%.

**Figure 2:**
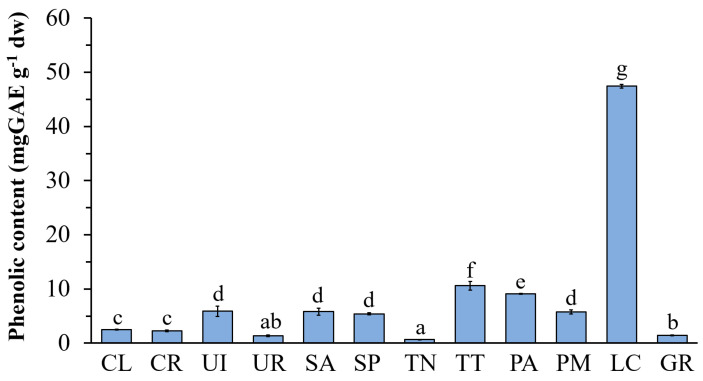
Total phenolic content (TPC) of the ethanolic extract from seaweed. The values are expressed as mean± SD (n = 3). Significant differences are indicated by different letters (*p* < 0.05).

The TPC varied significantly depending on species (Figure 2). The highest TPC was found in *L. challengeriae* (LC) (47.43 ± 0.35 mg GAE g^−1^ dw) for brown seaweed and in *U. intestinalis* (UI) (5.88 ± 0.35 mg GAE g^−1^ dw) for green seaweed. The TPC of *G. dura* was 1.45 ± 0.08 mg GAE g^−1^ dw.

### 3.2. Mushroom Tyrosinase Inhibitory Activity

**Figure 3:**
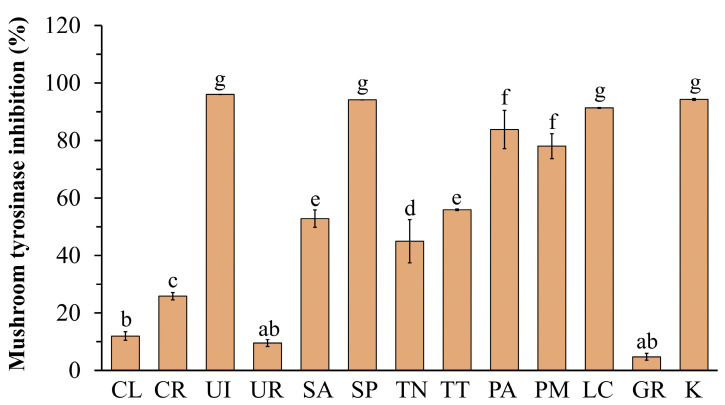
Effect of seaweed extracts and kojic acid (K) at 5 mg mL^−1^ on mushroom tyrosinase activity. The values are expressed as mean ± SD (n = 3). Significant differences are indicated by different letters (*p* < 0.05).

As shown in Figure 3, the mushroom tyrosinase inhibitory activities of *U. intestinalis* (96.04 ± 0.00%), *S. polycystum* (94.15 ± 0.00%), and *L. challengeriae* (91.34 ± 0.16%) showed the highest activity at 5 mg mL^−1^ and were comparable to those of kojic acid (94.30 ± 0.34%). Among the seaweed groups, the brown seaweed extracts showed the greatest tyrosinase inhibitory activity. The half maximal inhibitory concentration (IC_50_) values are shown in Table 2. A low IC_50_ value indicated that the sample had strong anti-tyrosinase activity. The ethanolic extract of *L. challengeriae* showed the highest mushroom tyrosinase inhibitory activity, with IC_50_ values of 0.15 ± 0.01 mg mL^−1^. This treatment showed a higher inhibitory effect on mushroom tyrosinase than kojic acid (IC_50_ = 0.35 ± 0.05 mg mL^−1^), a known tyrosinase inhibitor. In addition, a moderate correlation (*r* = 0.480) was found between the tyrosinase inhibitory activity of all ethanolic extracts and their phenolic content.

### 3.3. Cytotoxicity of Seaweed Extracts on B16F10 Cells

**Figure 4:**
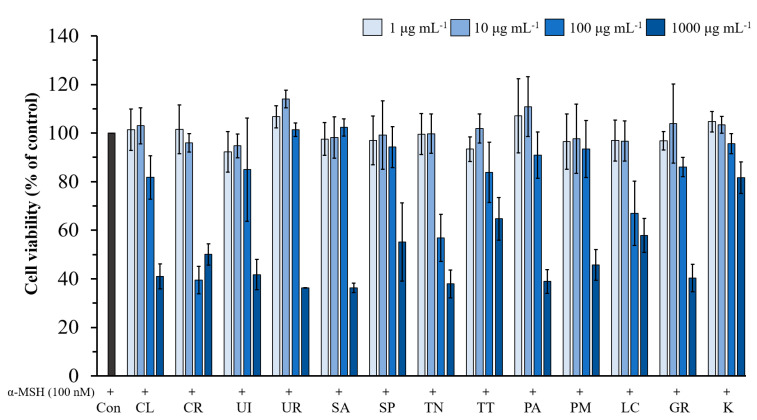
Effect of seaweed extract and kojic acid on cell viability in B16F10 melanoma cells. Cells were incubated with various concentrations (1–1000 µg mL^−1^) of seaweed extracts and kojic acid in the presence of α-MSH for 72 h. The values are expressed as mean ± SD (n = 3). Con; Control, K; Kojic acid.

The α-MSH stimulated B16F10 cells were treated with different concentrations of twelve seaweed extracts, and kojic acid (1–1000 µg mL^−1^) for 72 h (Figure 4). The results showed that none of the extracts had a significant effect on cell viability at concentrations of 1–10 µg mL^−1^. At 100 µg mL^−1^, some of the extracts exhibited cytotoxicity (<85% viable cells) and displayed significant toxicity (<75% viable cells) at 1000 µg mL^−1^, except kojic acid. Therefore, we used seaweed extract at a concentration of 10 µg mL^−1^ to determine its effect on melanin content in B16F10 cells.

**Figure 5:**
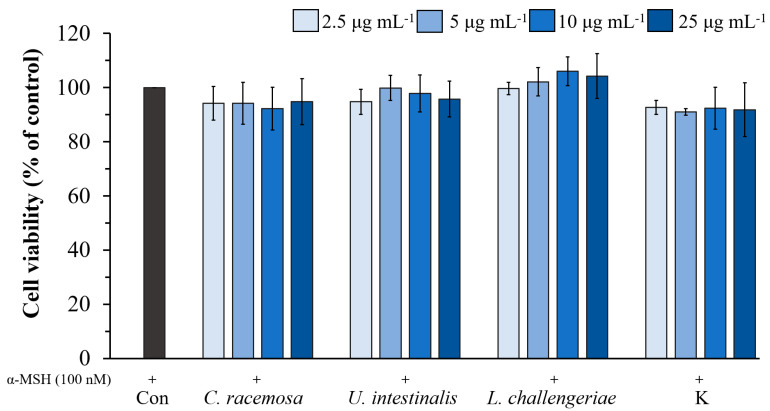
Effect of *C. racemosa*, *U. intestinalis*, and *L. challengeriae* on cell viability in α-MSH- stimulated B16F10 cells compared with kojic acid. Cells were incubated with various concentrations (2.5–25 µg mL^−1^) of seaweed extract and kojic acid for 72 h. The values are expressed as mean ± SD (n = 3). Con; Control, K; Kojic acid.

Later, the ethanolic extracts of *C. racemosa*, *U. intestinalis*, and *L. challengeriae* were selected to evaluate the effect of extract concentration on cell viability in B16F10 cells and compared with kojic acid. As shown in Figure 5, the cells treated with various concentrations (2.5–25 µg mL^−1^) of extracts from these three species exhibited more than 90% cell viability for up to 72 h.

### 3.4. Effects of Seaweed Extracts on Melanin Synthesis in B16F10 Cells

**Figure 6:**
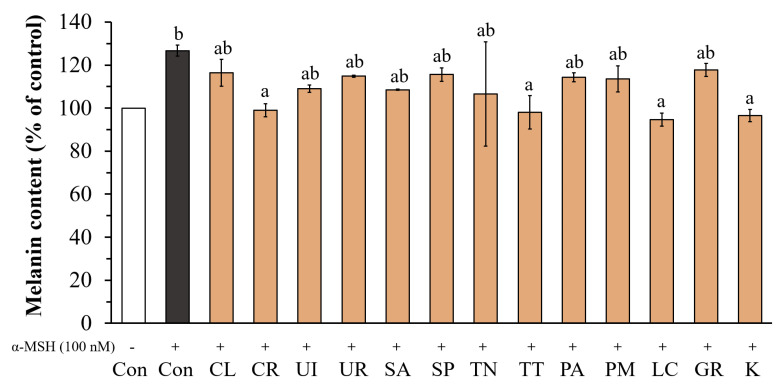
Effect of seaweed extracts and kojic acid at 10 µg mL^−1^ on cellular melanin content. The values are expressed as mean ± SD (n = 3). Significant differences are indicated by different letters (*p* < 0.05). Con; Control, K; Kojic acid.

The melanin contents of B16F10 cells were measured after treatment with twelve seaweed extracts at 10 µg mL^−1^ in the presence or absence of 100 nM α-MSH. As shown in Figure 6, all the seaweed extracts and kojic acid showed a significant inhibition of melanin production compared with the control stimulated by α-MSH. After the stimulation of B16F10 cells with α-MSH, the melanin content increased to 126.70% of the control (cells without α-MSH treatment). The ethanolic extracts of *C. racemosa* (CR), *T. conoides* (TT), and *L. challengeriae* (LC) showed maximal reductions in melanin production of 99.08 ± 3.04, 98.12 ± 7.74, and 94.67 ± 3.07%, respectively, which were not significantly different from that of kojic acid (96.55 ± 2.88%). In addition, a high correlation (*r* = 0.611) was found between the tyrosinase inhibitory activity of all ethanolic extracts and their phenolic content.

**Figure 7:**
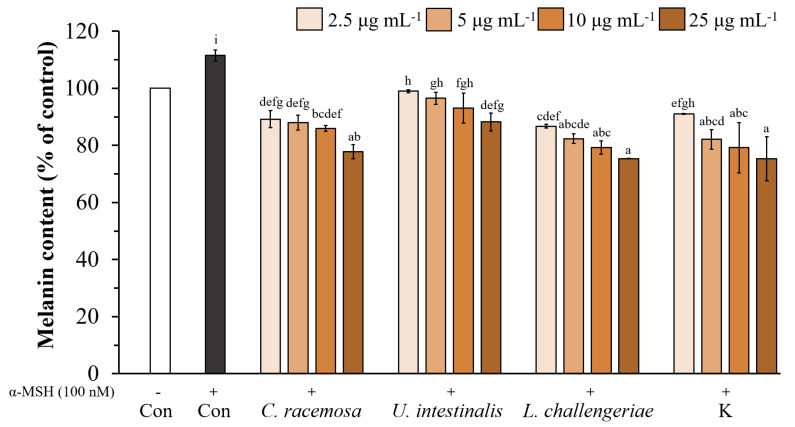
Effect of *C. racemosa*, *U. intestinalis*, and *L. challengeriae* on cellular melanin content in α-MSH-stimulated B16F10 melanoma cells compared with kojic acid. Cells were incubated with various concentrations (2.5, 5, 10, and 25 µg mL^−1^) of seaweed extract. The values are expressed as mean ± SD (n = 3). Significant differences are indicated by different letters (*p* < 0.05). Con; Control, K; Kojic acid.

**Figure 8:**
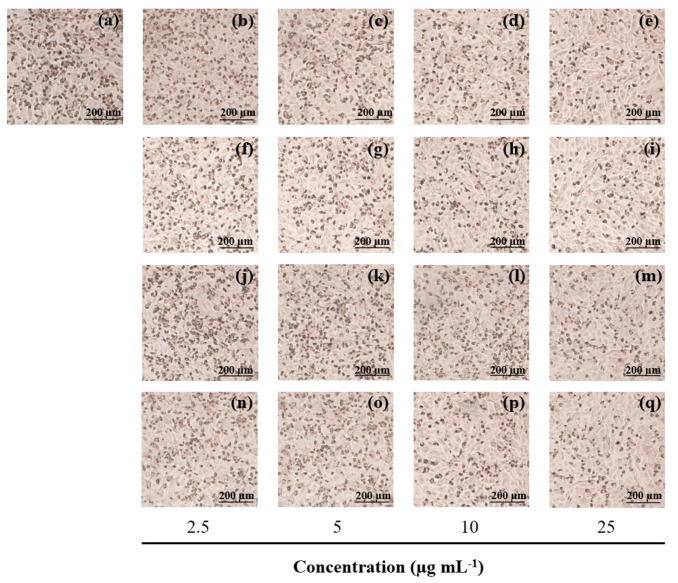
Effect of seaweed extracts and kojic acid on cellular melanin content in α-MSH-stimulated B16F10 melanoma cells. Cells were incubated with various concentrations of seaweed extract (2.5, 5, 10, and 25 µg mL^−1^) for 72 h. The melanin content (dark pigment) was observed under a microscope. Control in the presence of α-MSH (**a**), kojic acid (**b**–**e**), *C. racemosa* extract (**f**–**i**), *U. intestinalis* extract (**j**–**m**), *L. challengeriae* extract (**n**–**q**). Scale bar = 200 µm.

Moreover, when α-MSH-stimulated B16F10 cells were treated with various concentrations (2.5–25 µg mL^−1^) of the ethanolic extract of *C. racemosa*, *U. intestinalis*, *L. challengeriae*, and kojic acid, the melanin content of the cells decreased in a dose-dependent manner (Figure 7 and Figure 8). The melanin content of the α-MSH treated cells increased to 111.49% of the control cells (cells without α-MSH treatment). At 25 µg mL^−1^, the ethanolic extracts of *C. racemosa*, *U. intestinalis*, and *L. challengeriae* reduced melanin production in the α-MSH-stimulated cells to 77.78 ± 2.44%, 88.20 ± 3.09%, and 75.35 ± 0.05%, respectively. Moreover, the ability of *C. racemosa* and *L. challengeriae* to reduce melanin production was comparable to that of kojic acid (75.31 ± 7.71% at 25 µg mL^−1^).

### 3.5. Effects of Seaweed Extracts on Intracellular Tyrosinase Activity in B16F10 Cells

**Figure 9:**
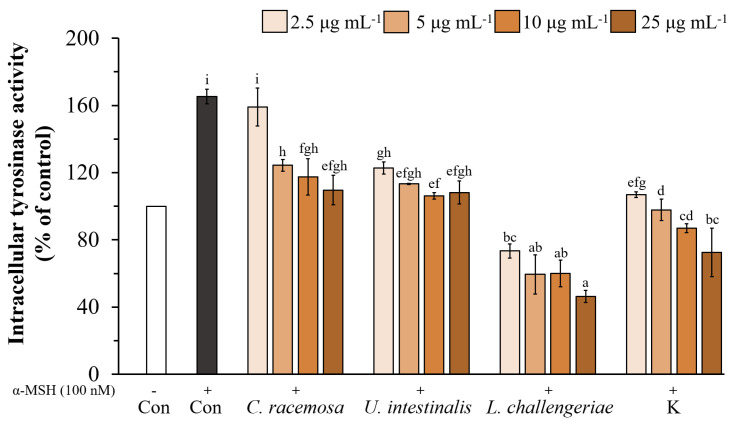
Effect of *C. racemosa*, *U. intestinalis*, and *L. challengeriae* on cellular tyrosinase activity in α-MSH-stimulated B16F10 melanoma cells compared with kojic acid. Cells were incubated with various concentrations (2.5–25 µg mL^−1^) of seaweed extract. The values are expressed as mean ± SD (n = 3). Significant differences are indicated by different letters (*p* < 0.05). Con; Control, K; Kojic acid.

We investigated the effect of the ethanolic extracts of *C. racemosa*, *U. intestinalis*, *L. challengeriae*, and kojic acid on cellular tyrosinase activity in α-MSH-stimulated cells. The cellular tyrosinase activity was significantly decreased by *C. racemosa*, *U. intestinalis*, *L. challengeriae*, and kojic acid in a dose-dependent manner (Figure 9). After the stimulation of B16F10 cells with α-MSH, intracellular tyrosinase activity increased to 165.23% of the control (cell without α-MSH treatment). At 25 µg mL^−1^, *C. racemosa*, *U. intestinalis*, and *L. challengeriae* reduced intracellular tyrosinase activity in the α-MSH-stimulated cells to 109.63%, 108.20%, and 46.30%, respectively. Moreover, *L. challengeriae* exhibited stronger inhibitory activity on intracellular tyrosinase than kojic acid (72.50% at 25 µg mL^−1^).

## 4. Discussion

Various skin-whitening agents made from natural or synthetic chemical substances are commercially available, but some agents have various side effects. Thus, further research is needed to explore natural whitening agents that are effective and safe for users to replace the chemicals currently used. Many studies have reported the skin-whitening effects of extracts and compounds isolated from natural sources [6,12,46,47]. Currently, many marine resources, especially seaweed, have attracted attention in the search for bioactive compounds for drugs and cosmetics. Numerous studies have reported the effects of seaweed extract on melanogenesis. Most of the studies focused on brown seaweed, such as *Ecklonia cava* Kjellman (1885) [9,22], *E. maxima* (Osbeck) Papenfuss (1940) [21], *Sargassum fusiforme* (Harvey) Setchell (1931) [10], *S. plagiophyllum* C. Agardh (1824) [18], *S. polycystum* [43,48,49], *Turbinaria conoides* [50], and *Undaria pinnatifida* (Harvey) Suringar (1873) [51]. Phlorotannins, fucoxanthin, and fucoidan from brown seaweed can inhibit melanogenesis in B16F10 melanoma cells [9,52,53]. Some studies have reported the anti-tyrosinase or anti-melanogenic activities of red seaweed, *Kappaphycus alvarezii* (Doty) Doty ex P. C. Silva (1996) [18,54], *Betaphycus gelatinus* (Esper) Doty ex P. C. Silva (1996) [55], *Gracilaria fisheri* (B. M. Xia and I. A. Abbott) I. A. Abbott, J. Zhang and B. M. Xia (1991) [23], *G. chouae* Zhang and B. M. Xia (1992), *G. blodgettii* Harvey (1853), *G. lemaneiformis* (Bory de Saint-Vincent) Greville (1830), *Gelidium amansii* (J. V. Lamouroux) J. V. Lamouroux (1813) and *Pyropia haitanensis* (T. J. Chang and B. F. Zheng) N. Kikuchi and M. Miyata (2011) [55], and *Symphyocladia latiuscula* (Harvey) Yamada (1941) [24]. The chemical components that respond to their activity are a group of sulfated galactans and bromophenols. For green seaweed, a relatively low inhibitory effect on mushroom tyrosinase has been reported from *C. racemosa* [56], *Ulva clathrata* (Roth) C. Agardh (1811), and *U. australis* Areschoug (1854) [19], *U. lactuca* Linnaeus (1753) [25], *Caulerpa* spp., and *Halimida* spp. [26]. However, no information is available about the anti-melanogenic activity in B16F10 melanoma cells of *C. racemosa*, *U. intestinalis*, and *L. challengeriae*.

In the present study, we investigated the anti-melanogenic potential of twelve seaweeds from the coastal area of Thailand, with a particular focus on the abundant species. Ethanol was used for extraction because it is known as a compatible solvent for polyphenol extraction, can extract a wide range of compounds that have a different polarity, and is considered a “greener” solvent [26,57]. The highest extraction yield was found in *L. challengeriae* (LC), higher than that of the ethanolic extract of *L. variegata* (J. V. Lamouroux) Womersley ex E. C. Oliveira (1977) (5.36%) reported by Zárate et al. [58]. Differences in yield can occur due to different environmental factors, such as the season, salinity, depth, size, age, and life cycle of seaweed [59,60,61].

Phenolic compounds are a group of secondary metabolites that have important functions in plants and seaweed. Recently, many researchers have reported the bioactivity of phenolic compounds [62]. Phenolic compounds are also the largest groups of tyrosinase inhibitors, including flavonoids and phlorotannins [6,7,22]. The results of this study showed that the highest level of TPC per seaweed dry weight was found in the Dictyotaceae species *L. challengeriae* (47.43 ± 0.35 mg GAE g^−1^ dw, 4.74% dw). Dictyotales are known for their high levels of TPC among brown seaweeds [26,61]. This result was consistent with Targett et al. [63], who reported that the TPC in *L. variegata* ranged from 4.27% to 25.47% dw. However, this study reported a lower TPC than that of *L. variegata* (29.18% dw) reported by Zubia et al. [64]. TPC varies according to numerous factors, such as species, season, age, geographical location, and environmental conditions. Numerous studies on brown algae have reported high TPC levels, characterized by extremely high biological activity and higher content than green and red algae [65,66,67]. Moreover, the higher TPC of brown seaweed may be caused by phlorotannins, olymers of phloroglucinol, which are found only in brown seaweed [68,69].

Many melanocyte-related enzymes are involved in melanogenesis, especially tyrosinase, the rate-limiting enzyme that plays an important role in melanin biosynthesis [6]. Thus, compounds that can inhibit tyrosinase activity are considered to have potential as anti-melanogenic agents. According to the effects on mushroom tyrosinase activity, the ethanolic extract of *L. challengeriae* showed a high inhibitory activity on tyrosinase with an IC_50_ of 0.15 ± 0.01 mg mL^−1^ and a relatively stronger tyrosinase inhibitory effect than kojic acid (IC_50_ = 0.35 ± 0.05 mg mL^−1^), a well-known tyrosinase inhibitor. Moreover, the ethanolic extract of *L. challengeriae* in this study had stronger tyrosinase inhibitory activity than the ethanolic extract of *L. variegata* (IC_50_ > 0.25 mg mL^−1^) reported by Zárate et al. [58] and *E. stolonifera* Okamura (1913) (IC_50_ = 0.345 mg mL^−1^) reported by Kang et al. [19]. However, for tyrosinase inhibitors in the literature, the IC_50_ values might be incomparable due to the varied assay conditions, including different substrate concentrations, different incubation times, and different lots of commercial mushrooms tyrosinase [6]. However, the cell-free mushroom tyrosinase inhibition assay is a commonly used screening method for potential skin-whitening. This assay is simple and inexpensive to use. However, the ability of the extracts to inhibit mushroom tyrosinase were not fully consistent with the results of in vivo depigmentation [49].

In this study, B16F10 melanoma cells were used as a model because they are commonly used in studies of melanogenesis and depigmentation. The cells can produce melanin by associating with intracellular tyrosinase activity, responding to stimulation by α-MSH, and exhibiting most melanogenic properties similar to those of normal human melanocytes [23]. Therefore, we examined the effect of twelve seaweed extracts on B16F10 cells. All seaweed extracts and kojic acid at 10 µg mL^−1^ showed a significant inhibition of melanin production compared with the control in the presence of α-MSH (Figure 6). The ethanolic extract of *L. challengeriae* (LC) showed the strongest ability to reduce melanin production (by 32.03%), followed by *T. conoides* (TT) and *C. racemosa* (CR), which reduced melanin production by 28.58 and 26.92%, respectively; these results were not significantly different from those of kojic acid (by 30.15%). These results are consistent with the results for 1,9-dihydroxy-crenulide and epiloliolide isolated from *Dictyota coriacea* (Holmes) I. K. Wang, H.-S. Kim and W. J. Lee (2004), at a concentration of 30 µg mL^−1^, which reduced melanin production by 27.8 and 22.6%, respectively [70].

As it showed the highest inhibition of mushroom tyrosinase activity and melanin production in B16F10 cells, *L. challengeriae* was selected for further study. Two other species of green seaweed, *U. intestinalis* and *C. racemosa*, were also selected for study because of the high anti-mushroom tyrosinase activity of *U. intestinalis* (96.04% at 5 mg mL^−1^) and the ability to reduce melanin synthesis in B16F10 cells of *C. racemosa* (26.92% at 10 µg mL^−1^). Furthermore, these two species can be cultivated on a large scale in a pond [49,71], which might be useful for future utilization. Then, we studied the effect of extract concentration on melanin production and intracellular tyrosinase activity in B16F10 melanoma cells of these three species. The results showed that *C. racemosa*, *U. intestinalis*, *L. challengeriae*, and kojic acid reduced melanin synthesis in the α-MSH-treated B16F10 cells in a dose-dependent manner. The ethanolic extracts of *C. racemosa* and *L. challengeriae* at a concentration of 25 µg mL^−1^ reduced melanin content by 33.71 and 36.14%, respectively, which was not significantly different (*p* < 0.05) from that of kojic acid (36.18%). However, the ethanolic extract of *U. intestinalis* reduced melanin content by 23.29%. In addition, *C. racemosa*, *U. intestinalis*, *L. challengeriae*, and kojic acid showed a significant inhibition of intracellular tyrosinase activity in the α-MSH-treated cells. The ethanolic extract of *L. challengeriae* showed a strong inhibitory effect on intracellular tyrosinase activity (reduced from 165.23% to 46.30%), which was significantly higher than that of kojic acid (to 72.50%).

The mushroom tyrosinase assay was used to determine the direct effect of seaweed extract on tyrosinase activity. The ethanolic extract of *C. racemosa* had a weak ability to inhibit mushroom tyrosinase (25.84 ± 1.27% at 5 mg mL^−1^). However, there was a dose-dependent decrease in intracellular tyrosinase activity in B16F10 melanoma cells after treatment with *C. racemosa* at concentrations of 2.5–25 µg mL^−1^. In addition, ethanolic extracts of *U. intestinalis*, *L. challengeriae*, and kojic acid inhibited both mushroom tyrosinase activity and intracellular tyrosinase activity. This result suggests that the inhibition of melanogenesis by *C. racemosa* extract does not directly inhibit tyrosinase activity, but may inhibit intracellular tyrosinase expression. The results with *C. racemosa* are consistent with the findings of the ethanolic extract of *S. polycystum* and sulfated galactan isolated from *G. fisheri*, which did not show an inhibitory effect on mushroom tyrosinase but reduced intracellular tyrosinase activity in B16F10 melanoma cells [23,43]. The commonly used mushroom tyrosinase assay still has some limitations in the application of the fungal tyrosinase inhibitor since fugal tyrosinase and human tyrosinase are different. Mushroom tyrosinase is a cytosolic enzyme, while melanocyte tyrosinase is membrane-bound enzyme; thus, the effect of anti-melanogenic agents on these tyrosinases may not be the same. Since the target cells of the agents are melanocytes, it would be more reliable to use tyrosinase derived from melanin-producing cells instead of fungi [6,43].

Melanin production in human skin is a complex process involving many enzymatic cascades, including those of tyrosinase, TRP-1, TRP-2, and their transcription factors, such as microphthalmia-associated transcription factor (MITF), cyclic adenosine monophosphate (cAMP) response element binding protein (CREB), and extracellular-regulated kinase (ERK) [5]. The agents that inhibit the expression and activity of those enzymes or proteins may have value as potential anti-melanogenic agents. It has been reported that extracts from seaweed, *G. fisheri*, and *Undaria pinnatifida* (Harvey) Suringar (1873) mediate the inhibition of melanogenesis in cells through the regulation of MITF [23,51]. The inhibitory effect of *Ishige foliacea* Okamura (1935) and *S. serratifolium* (C. Agardh) C. Agardh (1820) on α-MSH-stimulated melanogenesis via the extracellular signal-regulated kinase (ERK) signaling pathway has also been reported [72,73]. Therefore, the mechanism by which seaweed extracts inhibit MITF or the expression of other related proteins needs further study.

## 5. Conclusions

In the cosmetics industry, skin lighteners are often added to products. Most of them are substances that reduce melanin pigmentation by inhibiting the activity of the enzyme tyrosinase. However, some of the chemicals currently used have also shown dangerous side effects. Therefore, the idea of using natural extracts as substitutes has been raised. Seaweeds are an interesting resource, and several studies have reported the inhibition of tyrosinase activity and melanin production by their extracts.

The results of the present study show that the ethanolic extracts of *C. racemosa*, *U. intestinalis*, and *L. challengeriae* can inhibit mushroom tyrosinase activity and reduce melanin synthesis in α-MSH-stimulated B16F10 cells. These results suggest that the extracts of these algae are promising candidates for the development of safe pharmacological or cosmetic agents that have potent inhibitory effects on tyrosinase activity and melanin synthesis. However, the purification, structure, and mechanisms of the active components of these selected algae need further investigation.

## Figures and Tables

**Table 1:** List of seaweed collected during this study.

Sample Code	Species	Location	Collection Time	Voucher Number
Chlorophyta				
CL	*Caulerpa lentillifera* J. Agardh (1837) [35]	Chonburi Province (12°36′49.1″ N 100°55′26.2″ E)	March 2017	FIKU_119
CR	*Caulerpa racemosa* (Forsskål) J. Agardh (1873) [27]	Chonburi Province (12°36′49.1″ N 100°55′26.2″ E)	March 2017	FIKU_120
UI	*Ulva intestinalis* Linnaeus (1753) [28]	Pattani Province (6°52′39.4″ N 101°14′12.1″ E)	January 2017	FIKU_118
UR	*Ulva rigida* C. Agardh (1823) [36]	Phetchaburi Province (13°02′36.2″ N 100°05′11.0″ E)	August 2017	FIKU_127
Ochrophyta				
SA	*Sargassum aquifolium* (Turner) C. Agardh (1820) [36]	Chonburi Province (12°36′49.1″ N 100°55′26.2″ E)	May 2017	FIKU_124
SP	*Sargassum polycystum* C. Agardh (1824) [37]	Chonburi Province (12°36′10.0″ N 100°57′09.7″ E)	March 2017	FIKU_121
TN	*Turbinaria conoides* (J. Agardh) Kützing (1860) [38]	Chonburi Province (12°36′49.1″ N 100°55′26.2″ E)	May 2017	FIKU_125
TT	*Turbinaria coniodes* (J. Agardh) Kützing (1860) [38]	Trat Province (12°11′13.2″ N 102°18′04.6″ E)	March 2017	FIKU_122
PA	*Padina australis* Hauck (1887) [39]	Chonburi Province (12°36′10.0″ N 100°57′09.7″ E)	April 2017	FIKU_123
PM	*Padina minor* Yamada (1925) [40]	Trat Province (12°11′13.2″ N 102°18′04.6″ E)	December 2016	FIKU_117
LC	*Lobophora challengeriae* C. W. Vieira (2019) [29]	Chonburi Province (12°36′49.1″ N 100°55′26.2″ E)	May 2017	FIKU_126
Rhodophyta				
GR	*Gracilaria dura* (C. Agardh) J. Agardh (1842) [41]	Chonburi Province (13°12′30.0″ N 100°58′29.2″ E)	September 2016	FIKU_116

**Table 2:** The half-maximal inhibitory concentration (IC_50_) values of seaweed extract on mushroom tyrosinase activity.

Sample	IC_50_ (mg mL ^−1^)
*Caulerpa lentillifera* (CL)	>5.00
*Caulerpa racemose* (CR)	>5.00
*Ulva intestinalis* (UI)	3.35 ± 0.12
*Ulva rigida* (UR)	>5.00
*Sargassum aquifolium* (SA)	4.56 ± 0.14
*Sargassum polycystum* (SP)	1.24 ± 0.24
*Turbinalis coniodes* (TN)	>5.00
*Turbinalis coniodes* (TT)	4.62 ± 0.04
*Padina australis* (PA)	1.09 ± 0.03
*Padina minor* (PM)	1.23 ± 0.06
*Lobophora challengeriae* (LC)	0.15 ± 0.01
*Gracilaria dura* (GR)	>5.00
Kojic acid (K)	0.35 ± 0.05

Values are expressed as mean ± standard deviation (n = 3).

## Data Availability

Not applicable.
